# Accouchement du siège par voie basse: étude de la morbi-mortalité maternelle et néonatale

**DOI:** 10.11604/pamj.2014.17.27.2037

**Published:** 2014-01-17

**Authors:** Olivier Mukuku, Julien Kimbala, Justin Kizonde

**Affiliations:** 1 Département de Gynécologie-Obstétrique, Clinques Universitaires de Lubumbashi, RD Congo

**Keywords:** Présentation du siège, accouchement par voie basse, morbi-mortalité maternelle et néonatale, Breech presentation, vaginal delivery, maternal and neonatal morbidity and mortality

## Abstract

Le but de cette étude était de déterminer la fréquence de l'accouchement en présentation du siège aux Cliniques Universitaires de Lubumbashi, décrire les caractéristiques sociodémographiques et obstétricales en rapport avec les accouchées et évaluer la morbi-mortalité maternelle et néonatale liée à l'accouchement du siège par voie basse en comparaison avec l'accouchement du sommet par la même voie. Il s'agissait d'une étude rétrospective descriptive et analytique portant sur 31 accouchements par voie basse (VB), avec f'tus en présentation du siège, des grossesses monofoetales d’âge gestationnel supérieur à 35 semaines aménorrhées, réalisés au cours de la période allant du 1^er^ janvier 2010 au 30 juin 2011 à la maternité des Cliniques Universitaires de Lubumbashi en RD Congo. Les paramètres sociodémographiques, l'environnement obstétrical et l'issue maternelle et néonatale ont été analysés en comparaison avec ceux des 99 couples Mère-Enfant issus des accouchées eutociques (VB) au cours de la même période. Le seuil de signification a été fixé à p<,05. La fréquence de la présentation du siège est de 2,5% et le siège décomplété est la variété la plus retrouvée (60%). L’âge moyen, la parité moyenne, l’âge gestationnel moyen ainsi que le poids de naissance moyen sont comparables dans les 2 groupes (p=0,3308, p=0,6897, p=0,4420 et p=0,8240). La morbidité maternelle est caractérisée par un taux de 19,3% de lésions des parties molles parmi les accouchées avec foetus en présentation du siège contre 6,1% parmi les accouchées avec foetus en présentation du sommet (p=0,1197). La morbidité périnatale est représentée par un taux de dépression néonatale à la fin de la 1ère minute plus élevé chez les nouveau-nés en présentation du siège (38,7%) que ceux nés en présentation du sommet (5,1%) (p=0,0000) signifiant un risque de dépression néonatale multiplié par près de 12 (OR=11,87 3,35-44,51). A partir de la 5ème minute, le risque de dépression néonatale n'est pas différent quelque soit la présentation foetale considérée. S'agissant des déperditions néonatales, si aucun décès n'a été enregistré parmi les nouveau-nés en présentation du sommet, il a été déploré 2 décès parmi ceux nés en présentation du siège (6,5%; p=0,0554). Le séjour hospitalier moyen des accouchées ainsi que celui de leurs nouveau-nés sont comparables dans les deux groupes. La morbidité maternelle et néonatale observée dans l'accouchement du siège, matérialisée par un taux élevé des lésions périnéales et la dépression néonatale à la 1ère minute, est vraisemblablement le reflet du niveau des accoucheurs quant à la maîtrise des techniques dans la direction d'un accouchement du siège par VB. Il s'agit d'une morbidité non imputable à la seule présentation et donc totalement évitable.

## Introduction

La présentation du siège ne peut pas être considérée tout à fait comme une présentation normale. Seule la présentation du sommet répond à une accommodation parfaite du fœtus normal ayant un placenta normalement inséré, à l'utérus normalement constitué et de tonicité normale [1]. Elle représente 3 à 4% de l'ensemble des accouchements [1–3] et de ce fait, elle devrait constituer une préoccupation permanente dans la pratique quotidienne de l'obstétricien.

En effet, le mode de naissance par voie basse (VB) ou par voie haute (VH) reste encore débattu par de nombreuses études récentes [4, 5]. Certains auteurs plaident en faveur d'une césarienne systématique dans le cadre de ce type de présentation [6] et d'autres, à l'inverse, semblent en faveur d'une possible acceptation de la VB dans le cadre d'une sélection rigoureuse des patientes et ceci sans augmentation réelle de la morbi-mortalité néonatale mais avec un gain maternel considérable [7, 8]. Comparé à l'eutocie, l'accouchement du siège garde une mauvaise réputation, cependant, l'opération césarienne ne constitue pas obligatoirement la solution pour la terminaison de l'accouchement en cas de présentation du siège.

En effet, la césarienne s'accompagne d'une augmentation de la mortalité et de la morbidité maternelle et ne met pas à l'abri des complications fœtales asphyxiques ou traumatiques au cours de l'extraction. Un accouchement par VB, lorsque certaines règles sont respectées, donne d'aussi bons résultats [9].

Le présent travail s'inscrit dans une démarche de retracer l'influence de la présentation du siège sur le pronostic maternel et néonatal, en nous intéressant exclusivement aux grossesses monofœtales d’âge gestationnel supérieur à 35 semaines aménorrhées (SA) pour lesquelles toute contre-indication à l'accouchement par VB avait été éliminée. Ainsi, nous nous sommes fixés comme objectifs de déterminer la fréquence de l'accouchement en présentation du siège dans notre milieu, de décrire les caractéristiques sociodémographiques et obstétricales en rapport avec les accouchées et d’évaluer la morbi-mortalité maternelle et néonatale liée à l'accouchement du siège par VB en comparaison avec l'accouchement du sommet par la même voie.

## Méthodes

Il s'agit d'une étude rétrospective descriptive et analytique portant sur 31 accouchements par voie basse (VB), avec fœtus vivant en présentation du siège, des grossesses monofœtales d’âge gestationnel supérieur à 35 semaines aménorrhées (groupe I), réalisés au cours de la période allant du 1^er^ janvier 2010 au 30 juin 2011 à la maternité des Cliniques Universitaires de Lubumbashi en République Démocratique du Congo. Les paramètres sociodémographiques, l'environnement obstétrical et l'issue maternelle et néonatale ont été analysés en comparaison avec ceux des 99 couples Mère-Enfant issus des accouchées eutociques (groupe II) au cours de la même période.

Les données en rapport avec les paramètres sociodémographiques (âge maternel, parité), l'environnement obstétrical (âge gestationnel) et les résultats post-partals et néonatals ont été codifiées, puis saisies à l'ordinateur dans son langage Access et les analyses statistiques réalisées sur le logiciel Epi Info 2008 (version 3.5.1).

Les moyennes sont présentées avec les écarts-types (DS) et les Odds ratio (OR) avec un intervalle de confiance à 95% (IC à 95%: méthode de Cornfield). Le test de Student a été utilisé pour la comparaison des moyennes et celle des fréquences (exprimées en pourcentage) par le test de X^2^ corrigé de Yates ou le test exact de Fisher lorsque recommandés. Le seuil de signification a été fixé à p <0,05. Les ajustements ont été réalisés par la méthode de régression logistique.

## Résultats

### Fréquence et variétés de la présentation du siège

Sur un total de 2113 accouchées consécutivement enregistrées au cours de la période d’étude, nous avons répertorié 53 accouchées en présentation du siège et dont l’âge gestationnel était supérieur à 35 SA, soit une fréquence de 2,5%. La variété du siège a pu être précisée dans près de 65% des cas (20 sur 31) et le siège décomplété a été la variété la plus observée (12 cas soit 60%).

### Caractéristiques sociodémographiques et environnement obstétrical

L’âge maternel moyen est de 29,0 ans (DS=5,8) dans le groupe I allant de 16 à 45 ans contre 30,1 ans (DS=6,2) dans le groupe II allant de 17 à 46 ans (p=0,3308). Dans l'ensemble, un peu plus de quatre accouchées sur 5 ont un âge compris entre 18 et 35 ans dans les deux groupes. La parité va de 1 à 11 chez l'ensemble des accouchées. La parité moyenne est de 3,6 (DS=2,6) dans le groupe I et est de 3,4 (DS=2,3) dans le groupe II, les deux moyennes ne montrent pas de différence statistiquement significative (p=0,6897). Il en est de même de la comparaison des différentes tranches comme nous montre le Tableau 1. Quant à l’âge gestationnel, il varie entre 36 et 42,5 SA dans les deux groupes. L’âge gestationnel moyen est de 39,1 SA (DS=1,5) dans le groupe I contre 39,3 SA (DS=1,4) dans le groupe II (p=0,4420). De même, la comparaison des fréquences des grossesses à terme (âge gestationnel ‘38 SA) et celles des grossesses non à terme (âge gestationnel <38SA) entre les deux groupes ne montre pas de différence statistique significative (p=0,8779). En ce qui concerne le poids de naissance, il a varié entre 2000 et 4200 grammes dans le groupe I autour d'une moyenne de 3073 grammes (DS=552,2) et entre 2200 et 4300 grammes dans le groupe II avec une moyenne de 3262 grammes (DS=419,5) (p=0,8240). Par ailleurs, les nouveau-nés ayant pesé moins de 2500 grammes représentent 13% dans le groupe I contre 5,1% dans le groupe II (p=0,2113) et les nouveau-nés ayant pesé au moins 4000 grammes représentent 10% dans le groupe I contre 4% dans le groupe II (p=0,3523).

**Tableau 1:** Caractéristiques sociodémographiques et obstétricales

Paramètres	Groupe I		Groupe II		p
	n	%	n	%	
Age maternel					
<18 ans	1	3,2	2	2,0	0,5615
18-35 ans	25	80,7	83	83,8	0,8891
>35 ans	5	16,1	14	14,2	0,7753
	(n=31)		(n=99)		
**Parité**					
1	7	22,6	26	26,3	0,8613
2	7	22,6	17	17,1	0,6802
3-4	9	29,0	28	28,3	0,8828
≥5	8	25,8	28	28,3	0,9689
	(n=31)		(n=99)		
**Age gestationnel**					
<38 SA	5	16,1	15	15,2	0,8779
38-42 SA	25	80,7	80	80,8	0,8095
>42 SA	1	3,2	4	4,0	1,0000
	(n=31)		(n=99)		
**Poids de naissance (grammes)**					
**<2500**	4	13,3	5	5,1	0,2113
2500–2999	8	26,7	17	17,2	0,3740
3000–3499	11	36,7	42	42,4	0,7265
3500–3999	4	13,3	31	31,3	0,0880
≥ 4000	3	10,0	4	4,0	0,3523
	(n=30)		(n=99)		

SA: semaines d'aménorrhée

### Morbi-mortalité maternelle (Tableau 2)

L'on a enregistré 6 cas de lésions des parties molles (19,3%) parmi les accouchées du groupe I, il s'agit de 5 cas de déchirures périnéales et un cas de déchirure vaginale; 6 cas de déchirures des parties molles (6,1%) parmi les accouchées du groupe II, il s'agit de trois déchirures cervicales, 2 cas de déchirures périnéales et un cas de déchirure vaginale. L'analyse statistique ne montre pas de différence significative quant au taux global de complications entre les deux groupes (p=0,1197). La durée d'hospitalisation maternelle était uniformément de 3 jours chez les accouchées du groupe I contre 3 à 6 jours pour les accouchées du groupe II autour d'une moyenne de 3,1 (DS=0,51), la différence n'est pas statistiquement significative (p=0,3306). Signalons qu'aucun décès maternel n'a été déploré parmi les 130 accouchées.

**Tableau 2:** Morbi-mortalité maternelle et néonatale

	n	%	P	OR ajusté	IC à 95%
**1. Morbidité maternelle**					
**Lésions des parties molles**					
Groupe I	6	19,3	0,0606	3,72	[0,96-14,57]
Groupe II	6	6,1	-	1	-
Séjour hospitalier anormal*					
Groupe I	0	0,0	-	1	-
Groupe II	3	3,0	1,0000	0,00	[0,00-7,36]
2. Séjour néonatal anormal*					
Groupe I	0	0,0	-	1	-
Groupe II	1	1,0	1,0000	0,00	[0,00 - 60,89]
**3. Décès néonatal**					
Groupe I	2	6,5	0,0554	ind	-
Groupe II	0	0,0	-		

* Le séjour hospitalier était considéré anormal lorsqu'il allait au-delà de trois jours pour des raisons médicales évidentes

### Morbi-mortalité périnatale

Dépression néonatale (Tableau 3): *A la fin de la 1* ^*ère*^ *minute*. Le score d'Apgar a varié entre 2 et 9 chez les nouveau-nés du groupe I autour d'une moyenne de 6,96 (DS=1,70) contre 5 et 10 dans le groupe II avec une moyenne de 8,43 (DS=0,91). La comparaison de ces moyennes montre une différence statistique très significative (p=0,0000) en défaveur des nouveau-nés du groupe I. Aucun nouveau-né dans le groupe II n'est né dans un état de mort apparente contre 3,2% des nouveau-nés dans le groupe I mais la différence n'est pas statistiquement significative (p=0,2384). De manière générale, le taux de dépression néonatale (score d'Apgar <7) est de 38,7% dans le groupe I contre 5,1% dans le groupe II exprimant un risque de dépression néonatale multiplié par près de 12 pour les nouveau-nés du groupe I comparativement à ceux du groupe II (OR=11,87: 3,35-44,51). *A la fin de la 5* ^*ème*^ *minute*. Le score a varié entre 4 et 10 dans le groupe I autour d'une moyenne de 8,35 (DS=1,51) contre une variation de 7 à 10 avec une moyenne de 9,31 (DS=0,77) dans le groupe II. La comparaison de ces deux moyennes montre une différence statistique très significative (p=0,0000), par contre le risque de dépression néonatale chez les nouveau-nés des deux groupes n'est pas statistiquement différent (OR: ind, p=0,0554). *A la fin de la 10* ^*ème*^ *minute*. Le score d'Apgar à la fin de la 10^ème^ minute a varié entre 4 et 10 avec une moyenne de 8,93 (DS=1,43) dans le groupe I contre 7 et 10 avec une moyenne de 9,89 (DS=0,41) dans le groupe II. La comparaison de ces deux moyennes montre une différence statistique très significative en défaveur du groupe I (p=0,0000) bien que le risque de dépression néonatale ne soit pas significativement différent dans les deux groupes (OR: ind, p=0,0554).

**Tableau 3:** Score d'APGAR

Score d'APGAR	Groupe I		Groupe II		
	n	%	n	%	p
**A la fin de la 1** ^**ère**^ **minute**					
≤3	1	3,2	0	0,0	0,2384
4-6	11	35,5	5	5,1	0,0000
≥7	19	61,3	94	94,9	0,0000
**A la fin de la 5** ^**ème**^ **minute**					
≤3	0	0	0	0,0	-
4-6	2	6,4	0	0,0	0,0554
≥7	29	93,6	99	100	0,0554
**A la fin de la 10** ^**ème**^ **minute**					
≤3	0	0	0	0,0	-
4-6	2	6,4	0	0,0	0,0554
≥7	29	93,6	99	100	0,0554

**Séjour hospitalier néonatal:** Concernant le séjour hospitalier néonatal, il a été uniformément de 3 jours dans le groupe I contre 3 à 5 jours dans le groupe II autour d'une moyenne de 3,02 jours (DS=0,04) (p=0,5903) et le risque d'un séjour hospitalier allongé (>3 jours) n'est pas statistiquement plus élevé concernant les nouveau-nés du groupe II comparé au groupe I (OR=0,0 (0,00-60,89).

**Mortalité néonatale:** Aucun décès néonatal n'a été enregistré dans le groupe II, le taux de déperdition néonatale est de 6,5% chez les nouveau-nés du groupe I, l'analyse statistique ne relève pas de différence significative (p=0,0554)

## Discussion

La présente étude relève une fréquence de 2,5% des accouchements en présentation du siège. La fréquence de la présentation du siège varie essentiellement en fonction du terme de la grossesse. Elle s’établit autour de 3 à 4% à terme [1–3, 5]. Dans la littérature, la fréquence globale de la présentation du siège varie très peu d'un auteur à un autre. La fréquence relevée dans cette étude se rapproche de 2,3% et 2,1% retrouvées respectivement par Delotte entre 1996 et 2005 au CHU de Nice en France [5] et Abha entre 2007 et 2009 en Inde [10]. Buambo-Bamanga en 2006 à Brazzaville et Thera à Mopti (Mali) de 2007 à 2009 ont rapporté des taux voisins de 4,7% et 3,6% [11, 12]. Rietberg, entre 1995 et 1999 en Hollande et Sy en 2006 en Guinée Conakry ont rapporté les fréquences des plus élevées qui sont respectivement de 8,2% et de 9,6% [13, 14].

La comparaison entre les différents auteurs nous parait difficile dans la mesure où tous n'ont pas utilisé les mêmes critères de recrutement. En effet, certains auteurs avaient recensé l'accouchement du siège dans les grossesses monofœtales à terme après exclusion des morts foetales in utero [13]. D'autres auteurs avaient inclus les grossesses âgées d'au moins 28 SA [10, 15].

La répartition des variétés de présentation à l'admission était la suivante: 60% étaient des sièges décomplétés et 40% étaient des sièges complets. Cette prédominance du siège décomplété est rapportée par plusieurs auteurs qui ont trouvé des résultats comparables aux nôtres: en France, Broche dans sa série entre 1988 et 2003 au CHU de Besançon a trouvé 64,2% de siège décomplété [16] et 72,6% pour Descargues entre 1993 et 1999 au CHU de Rouen [17]. Une étude menée par Buambo-Bamanga entre janvier 2001 et décembre 2002 à Brazzaville (République du Congo) révèle 65,1% des sièges décomplétés [11]. Vers le 7^ème^ mois de la grossesse, il y a mutation spontanée du fœtus en siège selon la loi d'adaptation de Pajot (adaptation du contenu au contenant) et à la suite de la modification de forme de l'utérus liée à la formation du segment inférieur. Au cours de cette mutation, suite à la pesanteur et à certains facteurs (hypotonicité utérine chez la multipare, hypoplasie utérine chez la nullipare, etc.), il en résulte un échec de la culbute physiologique pouvant expliquer ainsi la prédominance du siège décomplété (mode des fesses) retrouvée aussi bien dans la littérature que dans notre étude [2].

L’âge maternel moyen dans le groupe des accouchées avec fœtus en présentation du siège est de 29,±5,8 ans avec des extrêmes de 16 et 45 ans. Il est comparable à ceux trouvés dans les travaux de certains auteurs: 29 ans pour Raudrant [18] et 29,4 ans avec des extrêmes de 14 et 43 ans pour Buambo-Bamanga [11]. Par contre, d'autres ont trouvé un âge maternel moyen légèrement inférieur au nôtre: Nayama rapporte 24,5 ans (extrêmes de 15 et 50 ans) [15], Descargues lui trouve 26,7 ans (extrêmes de 17 et 47 ans) [17] et pour Broche, la moyenne d’âge maternel est de 28,6 ans [16]. Les différences d’âges maternels observées dans la littérature seraient tout simplement le reflet des différences dans les fourchettes des périodes d'activité sexuelle selon les milieux.

La comparaison d’âges moyens des accouchées et celle des tranches d’âge ne montre pas de différence entre les accouchées en présentation du siège et celles en présentation de sommet. Le risque de présentation du siège ne serait donc pas lié à l’âge maternel.

La parité moyenne est de 3,6±2,6 avec des extrêmes allant de 1 à 11 dans le groupe des accouchées en présentation du siège. La moyenne de parité ainsi que les différentes classes de parité dans le groupe des accouchées en présentation du siège ne sont pas différentes de celle des accouchées en présentation de sommet. Cette différence pourrait s'expliquer par le comportement pronataliste qui caractérise la société congolaise. Notre taux de primipares de 22,6% est proche de celui rapporte par Broche (20,8%) dans son étude [16] mais inférieur à 52,6% trouvé par Vistad [9].

L’âge gestationnel moyen dans le groupe des présentations du siège est de 39,1±1,5 SA et a varié entre 36 et 42,5 SA; il n'est pas statistiquement différent de l’âge gestationnel moyen chez les accouchées en présentation de sommet (39,3 SA). Ces résultats sont proches de ceux de Vistad et Broche qui ont trouvé respectivement un âge gestationnel moyen de 39,4 et 39,5 SA [9, 19, 20]. Ces similitudes s'expliqueraient tout simplement par les critères d'inclusion de notre étude comme chez les autres, ayant écarté les grossesses d’âge gestationnel inférieur à 36 SA.

Dans le groupe de présentation du siège, le poids moyen de naissance est de 3073±552,2 grammes et a varié entre de 2000 et 4200 grammes. Septante-six virgule six pourcent des nouveau-nés ont un poids compris entre 2500 et 3999 grammes. La moyenne pondérale ainsi que la distribution des différentes tranches pondérales ne sont pas différentes dans les 2 groupes étudiés. Ces résultats sont comparables à ceux de Broche qui a trouvé un poids moyen de 3081 grammes [19] et 3164 grammes pour Raudrant [18]. Buambo-Bamanga, dans sa série, relève que 63,5% des nouveau-nés ont un poids compris entre 2500 et 3999 grammes [11]. Ces concordances s'expliqueraient par nos critères d'inclusion qui ne considèrent que les grossesses âgées d'au moins 36 SA et à cet âge, le poids de naissance est d'au moins 2000 grammes. Par ailleurs, que le poids de naissance moyen soit cliniquement plus élevé chez les nouveau-nés en présentation de sommet est tout à fait compréhensible car la macrosomie ne fait appel à la VH qu'après échec de l’épreuve du travail dans nos habitudes alors qu'elle est directement sanctionnée par la césarienne en cas de présentation du siège.

Ni l’âge maternel, ni la parité, ni l’âge gestationnel, ni même le poids de naissance n'ont influé sur la présentation.

Dans la morbidité maternelle, nous avons noté 19,3% de lésions des parties molles parmi les accouchées du groupe I, lésions représentées par cinq cas de déchirures périnéales et un cas de déchirure vaginale; contre 6,1% de lésions des parties molles chez les accouchées du groupe II dont trois cas de déchirures cervicales, deux cas de déchirures périnéales et un cas de déchirure vaginale (p=0,1197). Sy trouve 26,5% des lésions périnéales dans le groupe des accouchées en présentation du siège contre 10,7% dans le groupe des accouchées en présentation de sommet (p=0,0000) [14]. Ces déchirures font généralement suite à des accouchements malconduits avec des extractions brutales du foetus, témoins de l'inexpérience du personnel accoucheur alors qu'elles pouvaient être évitées par la pratique systématique de l’épisiotomie.

En ce qui concerne la mortalité maternelle, aucun décès maternel n'a été enregistré durant notre étude. Il en est de même pour Nayama [15]; par contre, Buambo-Bamanga enregistre un taux de mortalité maternelle de 0,3% chez les accouchées avec fœtus en présentation du siège [11].

Comparés à ceux nés par le sommet, les nouveau-nés par siège ont présenté un taux significativement plus élevé de dépression néonatale à la première minute de naissance mais l’état de bien-être n'a pas montré de différence entre les deux groupes à la fin de la 5^ème^ et de la 10^ème^ minutes. Thera, dans une série de 92 sièges nés comparés à 184 sommets, ne trouve aucune différence à la première minute entre les deux groupes (p=0,63) [12]. Dans une étude finlandaise de 2004 où ont été étudié 1270 sièges nés par VB, comparés à plus de 130000 enfants nés par VB en présentation céphalique ou par césarienne, les auteurs concluent que pour les sièges par VB on note un score d'Apgar souvent inférieur dans les premières minutes de vie mais qu'il n'existe pas plus de morbidité sévère dans cette population que dans les autres [21]. Enfin, il en est de même pour Descargues qui signale qu'aucun score d'Apgar <7 à la 5^ème^ minute n'a été observé dans sa série [17]. Le taux élevé des dépressions néonatales à la naissance serait imputable au degré de qualification du personnel accoucheur en rapport avec la surveillance intrapartale que la conduite de l'accouchement proprement dit.

En outre, l’étude relève que l'accouchement en présentation du siège est greffé d'un taux élevé de décès néonatals précoces (6,5%) comparativement à celui en présentation de sommet (0%) (p=0,0554). Ce taux est proche de ceux rapportés par Buambo-Bamanga et Sy qui ont trouvé respectivement 7,8% et 7,6% [11, 14]. En Europe, beaucoup d'auteurs ont rapporté des taux inférieurs à 1%: Broche rapporte 0,9% [16]; en 1997, Lindqvist analyse 6542 accouchements par le siège du registre Suédois des naissances et trouve une mortalité de 0,09% pour les accouchements du siège par VB [22], et au cours de cette même année, l’équipe norvégienne d'Albrechsten, dans une série prospective de 1212 sièges, ne rapporte aucun décès néonatal [23]. Ces différences observées entre les pays nantis et les pays à faible pénétration sanitaire s'expliqueraient par la qualité de surveillance intrapartale, le degré de qualification du personnel accoucheur ainsi que la réanimation néonatale.

## Conclusion

Outre la dépression néonatale traduite par un score d'Apgar déprimé à la fin de la première minute, l'accouchement par le siège n'a pas montré une morbidité maternelle et néonatale exceptionnellement plus élevée comparé à l'eutocie bien que dans les deux cas la mortalité néonatale soit très élevée lorsque comparée aux résultats occidentaux. Avec un personnel spécifiquement bien formé, la morbi-mortalité périnatale qu'accompagne l'accouchement du siège pourrait être améliorée.
